# Phylogenetic relationship and characterization of the complete chloroplast genome of *Panax notoginseng*, the endemic medicinal herbs to China

**DOI:** 10.1080/23802359.2019.1623109

**Published:** 2019-07-10

**Authors:** Kangyu Wang, Honghua Sun, Chenxi Huang, Shaokun Li, Yi Wang

**Affiliations:** a College of Life Science, Jilin Agricultural University, Changchun, China;; b Research Center for Ginseng Genetic Resources Development and Utilization, Jilin Province, Changchun, China

**Keywords:** *Panax notoginseng*, chloroplast genome, medicinal herbs, phylogenetic relationship

## Abstract

*Panax notoginseng* is the most important valued and endemic medicinal herb to China. It belongs to the Araliaceae family, which has a longer medical history in China. The complete chloroplast genome of *Panax notoginseng* is 156,387 bp in size and displays a typical quadripartite structure of the large single-copy region (LSC, 86,112 bp), small single-copy region (SSC, 18,005 bp) that separate by a pair of inverted repeat regions (IRs, each for 26,135 bp). The base nucleotide composition of the cpDNA is 30.8% of A, 31.1% of T, 19.9% of C, and 18.2% of G, with a total G + C content of 38.1%. The whole chloroplast genome of *P. notoginseng* contains 134 genes, including 89 protein-coding genes (PCGs), 37 transfer RNA (tRNAs) genes, and eight ribosomal RNA (rRNAs) genes species. Phylogenetic relationship analysis based on 37 medicinal herbs species confirmed the position of *P. notoginseng* closely related to *Panax japonicus* and *Panax vietnamensis*.

*Panax notoginseng* is the most important endemic medicinal herb to China and named “Southern God grass” by the old people. It is mainly grown (85%) in Yunnan province of China, which has been cultivated for more than 400 years and used as top-class medicine about 2000 years in China (Briskin 2000; Liu et al. 2015). Saponins have been found the main bioactive compounds in *P. notoginseng*, which have many biological activities, such as immunosti-mulating, hypocholesterolemic, anti-carcinogenic and anti-microbial (Ng 2006). *P. notoginseng* also shares many similar chemical constituents of saponins with *P. ginseng* and *P. quinquefolius* (Qiao et al. 2018). Research of *P. notoginseng* complete genome is very important for the Panax genus medicinal herbs in the world. In this study, we research the chloroplast genome of *P. notoginseng* and discuss genetic and phylogenetic relationship with other medicinal herbs, which provide more data information for study of the endemic medicinal herbs plants in evolution to China.

The specimen of *Panax notoginseng* was purchased from Tonghua town in Tonghua, Jilin, China. The total genomic DNA of *P. notoginseng* was extracted using CTAB method from the whole plant and stored at Jilin Agricultural University College of Life Science in −80 °C super cold refrigerator (Name No. JLAUCLS9). The cpDNA was purified and fragmented using the NEB Next Ultra™ II DNA Library Prep Kit (NEB, BJ and CN). Quality control was performed and removed low-quality reads using the FastQC software (Andrews 2015). The complete chloroplast genome of *P. notoginseng* was assembled using the MITObim version 1.8 (Hahn et al. 2013) and annotated using the DOGMA (Dual Organellar GenoMe Annotator) web (Wyman et al. 2004), with default settings to identify protein-coding genes, rRNAs and tRNAs based on the Plant Plastid Code and BLAST searches. The chloroplast (cp) genome map of the *P. notoginseng* chloroplast genome was generated using OGDRAW (Lohse et al. 2013).

The whole chloroplast genome sequence of *P. notoginseng* (NCBI No.MK6057261) is a closed-circular molecule of 156,387 bp in length, which is almost the same with the *P. quinquefolius* chloroplast (NC_027456.1 and 156,088 bp) and *P. ginseng* chloroplast (NC_006290.1 and 156,318 bp). The chloroplast genome of *P. notoginseng* has a large single-copy region (LSC) of 86,112 bp, a small single- copy region (SSC) of 18,005 bp and a pair of inverted repeat regions (IRs) of 26,135 bp. It comprised 134 functional genes which observed in this medicinal herb cpDNA, including 89 PCGs, 37 tRNA genes (four each for Isoleucine and Leucine, three each for Valine, Arginine and Serine, two each for Alanine, Asparagine, Glycine, Threonine and Methionine, one for each amino acid) and 8 genes for ribosomal RNA subunits (two each for *rrn*16, *rrn*23, *rrn*4.5, and *rrn*5). In the IR regions, a total of 20 genes were found duplicated, including nine PCGs species (*rps19, rpl2, rpl23, ycf2, ycf15, ndhB, rps7, rps12* and *ycf1*), seven tRNA genes species (*trnI-CAU, trnL-CAA, trnV-GAC, trnI-GAU, trnA-UGC, trnR-ACG,* and *trnN-GUU*), and four rRNA genes species (*rrn16, rrn23, rrn4.5,* and *rrn5*). The base composition of the cpDNA is as follows: 30.8% A, 31.1% T, 19.9% C, and 18.2% G, with a total AT content of 61.9% and GC content of 38.1%.

To study the phylogenetic relationship of *P. notoginseng*, the phylogenetic tree was used the Neighbour-Joining (NJ) methods and selected 36 medicinal herbs species published complete chloroplast genomes from GenBank to assess the genetic and phylogenetic relationship with *P. notoginseng*. The genome-wide alignment of 37 medicinal herbs species complete chloroplast genomes was carried out by HomBlocks (Bi et al. 2018). NJ analysis was performed using MEGA X (Kumar et al. 2018), which the bootstrap values were calculated using 5000 replicates to assess node support and all the nodes were inferred with strong support by the NJ methods. The phylogenetic NJ tree was constructed using MEGA X (Kumar et al. 2018) and edited using iTOL web server (https://itol.embl.de/). As shown in the phylogenetic NJ tree result (Figure 1), that the chloroplast genome of *Panax notoginseng* is clustered and closest with *Panax japonicus* (No.KP036469.1) and *Panax vietnamensis* (No.KP036471.1), also clustered closely to *P. ginseng* and *P. notoginseng*. The complete chloroplast of *P. notoginseng* provides more molecular data for the genetic diversity conservation.

**Figure 1.**
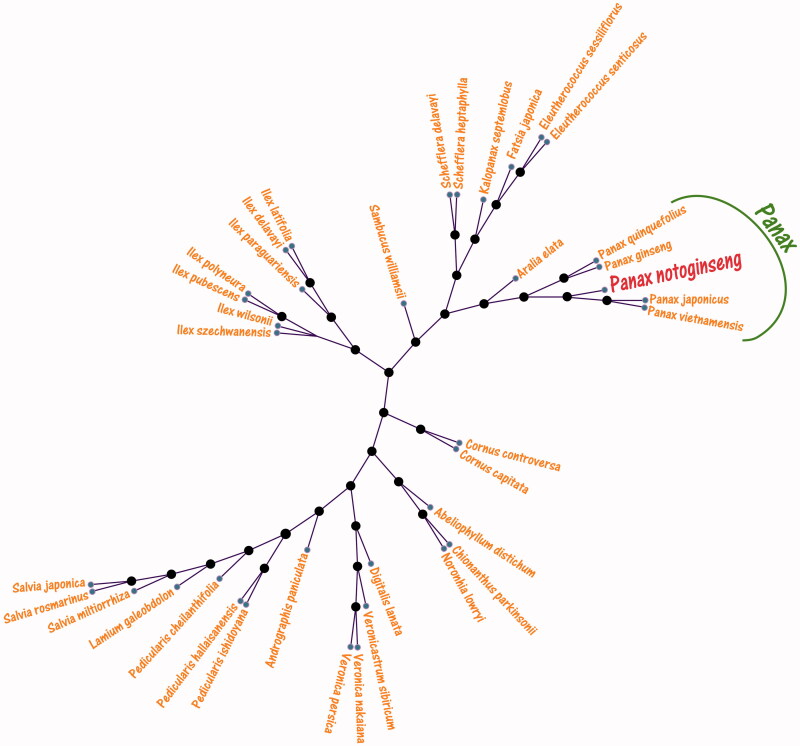
The Neighbour-Joining (NJ) phylogenetic tree from 36 medicinal herbs species chloroplast genomes with *Panax notoginseng*. All nodes exhibit above 90% bootstraps. The length of branch represents the divergence distance. 36 medicinal herbs species chloroplast genomes have been deposited in the GenBank, the accession numbers are as follows: *Abeliophyllum distichum* KT274029.1, *Andrographis paniculata* KF150644.2, *Aralia elata* KT153023.1, *Chionanthus parkinsonii* MG255752.1, *Cornus capitata* MG524998.1, *Cornus controversa* MG525004.1, *Digitalis lanata* KY085895.1, *Eleutherococcus senticosus* JN637765.1, *Eleutherococcus sessiliflorus* KT153019.1, *Fatsia japonica* KR021045.1, *Ilex delavayi* KX426470.1, *Ilex latifolia* KX426465.1, *Ilex paraguariensis* KP016928.1, *Ilex polyneura* KX426468.1, *Ilex pubescens* KX426467.1, *Ilex szechwanensis* KX426466.1, *Ilex wilsonii* KX426471.1, *Kalopanax septemlobus* KC456167.1, *Lamium galeobdolon* KY562590.1, *Noronhia lowryi* MG255759.1, *Panax ginseng* NC_006290.1, *Panax japonicus* KP036469.1, *Panax quinquefolius* NC_027456.1, *Panax vietnamensis* KP036471.1, *Pedicularis cheilanthifolia* KY751712.1, *Pedicularis hallaisanensis* MG770330.1, *Pedicularis ishidoyana* KU170194.1, *Salvia japonica* KY646163.1, *Salvia miltiorrhiza* HF586694.1, *Salvia rosmarinus* KR232566.1, *Sambucus williamsii* KX510276.1, *Schefflera delavayi* KC456166.1, *Schefflera heptaphylla* KT748629.1, *Veronica nakaiana* KT633216.1, *Veronica persica* KT724052.1, and *Veronicastrum sibiricum* KT724053.1.
